# Artificial intelligence facilitates the potential of simulator training: An innovative laparoscopic surgical skill validation system using artificial intelligence technology

**DOI:** 10.1007/s11548-024-03253-5

**Published:** 2024-08-19

**Authors:** Atsuhisa Fukuta, Shogo Yamashita, Junnosuke Maniwa, Akihiko Tamaki, Takuya Kondo, Naonori Kawakubo, Kouji Nagata, Toshiharu Matsuura, Tatsuro Tajiri

**Affiliations:** 1 Department of Pediatric Surgery, Reproductive and Developmental Medicine, Faculty of Medical Sciences, Kyushu University, 3-1-1 Maidashi, Higashi-ku, Fukuoka, 812-8582 Japan; 2 Exawizards Inc., Tokyo, Japan

**Keywords:** Laparoscopic surgery, Simulator training, Surgical education, Artificial intelligence, DeepLabCut

## Abstract

**Purpose:**

The development of innovative solutions, such as simulator training and artificial intelligence (AI)-powered tutoring systems, has significantly changed surgical trainees’ environments to receive the intraoperative instruction necessary for skill acquisition. In this study, we developed a new objective assessment system using AI for forceps manipulation in a surgical training simulator.

**Methods:**

Laparoscopic exercises were recorded using an iPad®, which provided top and side views. Top-view movies were used for AI learning of forceps trajectory. Side-view movies were used as supplementary information to assess the situation. We used an AI-based posture estimation method, DeepLabCut (DLC), to recognize and positionally measure the forceps in the operating field. Tracking accuracy was quantitatively evaluated by calculating the pixel differences between the annotation points and the points predicted by the AI model. Tracking stability at specified key points was verified to assess the AI model.

**Results:**

We selected a random sample to evaluate tracking accuracy quantitatively. This sample comprised 5% of the frames not used for AI training from the complete set of video frames. We compared the AI detection positions and correct positions and found an average pixel discrepancy of 9.2. The qualitative evaluation of the tracking stability was good at the forceps hinge; however, forceps tip tracking was unstable during rotation.

**Conclusion:**

The AI-based forceps tracking system can visualize and evaluate laparoscopic surgical skills. Improvements in the proposed system and AI self-learning are expected to enable it to distinguish the techniques of expert and novice surgeons accurately. This system is a useful tool for surgeon training and assessment.

## Introduction

Compared with open surgery, laparoscopic surgery has several advantages, including lower postoperative pain and infection, quicker recovery, and cosmetic benefits. It has become the gold standard for several procedures [1–3]. A wide variation in surgical skills among practitioners is associated with adverse intraoperative and postoperative patient outcomes. Training is essential for young surgeons to facilitate safe laparoscopic surgery. Good laparoscopic skills can be acquired through simulation training, which has proven effective in acquiring and transferring technical skills to the operating room [4–6]. Our group developed a disease-specific laparoscopic training simulator with an objective surgical skill evaluation system [7, 8] and verified the learning effect of continuous surgical training using this simulator [9]. However, subjective and experiential guidance are still used in actual clinical practice. The COVID-19 pandemic changed the opportunities for surgical trainees to receive the intraoperative instruction needed for skill acquisition [10], and innovative solutions, such as simulator training and artificial intelligence (AI)-powered tutoring systems, may help address such disruptions [11].

We developed a new objective evaluation system for forceps manipulation in a training simulator and verified the feasibility of developing and implementing a tool to quantify forceps movements in the training simulator.

## Methods

### Training simulator

In this training, we used the Laparoscopyboxx Pro (Laparoscopyboxx, Nijmegen, the Netherlands), a pediatric box trainer available on the market. This training box is made of wood and has the following parts: five Laparoscopyboxx ports, standard Laparoscopyboxx legs, pediatric Laparoscopyboxx legs, exercise boards, and a suturing pad. We attached an iPad® (Apple, Cupertino, CA) to the box and recorded each training procedure. Subsequently, we set the curved needle holder in the right hand and the Maryland forceps in the left hand.

### Participants

Ten randomly selected pediatric surgeons participated in this training. All participants were right-handed, provided informed consent, and voluntarily agreed to participate in this study. The participants were trained on needle trail, ring transfer, and suturing exercises in this training box.

### Method for tracking forceps movement

The procedure was recorded from top and side views using an iPad®. Although smartphones with high-performance cameras or DSLR cameras can be used, we selected the iPad because of its larger screen, which offers better visibility when providing real-time or near-real-time feedback to users. Top-view movies were used for AI forceps trajectory learning, whereas side-view movies were not directly used for learning because they required monocular tracking. However, they were used as supplementary information to assess the situation. The needle trail exercise video was used for basic AI learning. To recognize and positionally measure the forceps in the operating field, we used an AI-based posture estimation method known as DeepLabCut (DLC) [12]. Originally developed for behavioral studies in animals, such as mice and monkeys, DLC has been used to quantify the movements of various body parts (e.g., fingers and tails). This method has gained widespread use across various research sectors, particularly biological behavior analysis and medicine. DLC uses a training dataset, which requires a process known as labeling. Labeling is a crucial step in training the AI to identify and track-specific elements of interest. This process involves extracting multiple frames (or images) from the video footage and pinpointing particular sections of the object of interest within these frames. Each identified and labeled point within this process is referred to as a *keypoint*.

First, tracking accuracy was quantitatively evaluated by calculating the pixel difference between the annotation point and the point predicted by the AI model using needle trail exercises. Second, as a qualitative evaluation of the AI model, we verified tracking stability at key points (i.e., the tip of the forceps, hinge, and stick) using needle trail exercises. Furthermore, to evaluate the scalability of the AI model, we verified the tracking stability when the backgrounds were different using ring transfer and suturing exercises.

## Results

### Quantitative evaluation

**Fig. 1:**
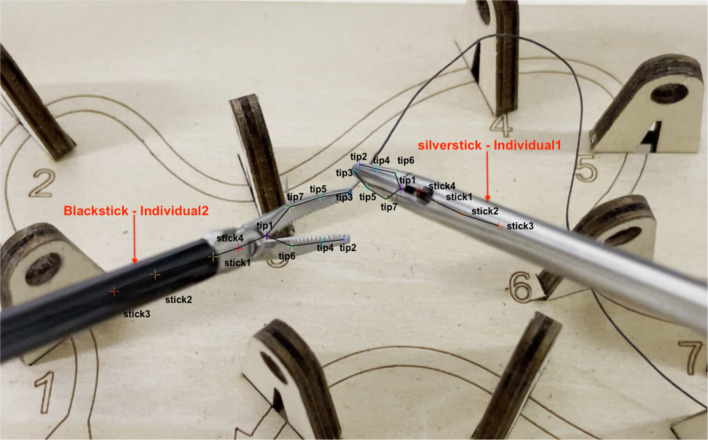
Labeled keypoints

**Fig. 2:**
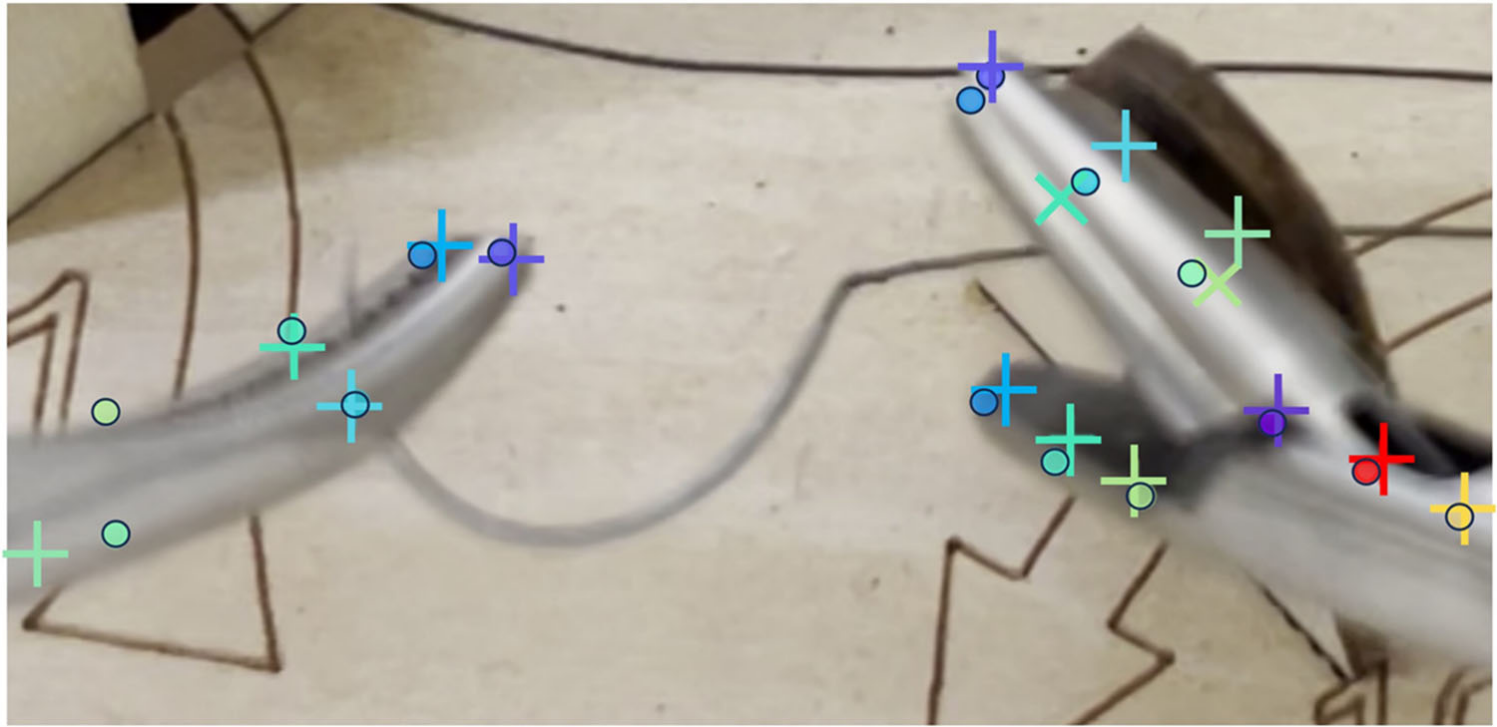
Comparison of the labeled and detected keypoints. Each component of the forceps, identified by DeepLabCut, is represented by a circular symbol (•), and labeled keypoints are marked by a cross symbol (+)

**Fig. 3:**
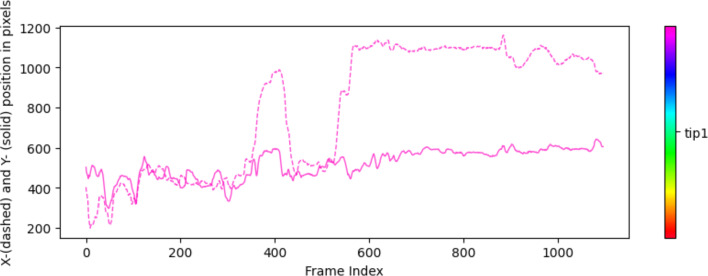
Tracking results with the curved needle holder. *X* is the lateral direction, and *Y* is the longitudinal direction

A detailed examination of keypoints denoted as *tip1* through *tip7* and *stick1* through *stick4* (Fig. 1) was conducted. These keypoints were labeled across a sequence of 300 frames. Figure 2 shows a comparison of the labeled and detected keypoints. Each component of the forceps, identified by DLC, is represented by a circular symbol (•), and labeled keypoints are marked by a symbol (+). Cross symbols (x) indicate points that were considered false positives and were excluded from the tracking process. These false positives encompass points initially recognized by DLC as components of the forceps but were identified as false positives upon further examination because of low confidence scores. This metric signifies the reliability of the detection process. Figure 3 shows the tracking results for the two types of forceps. *X* and *Y* indicate the pixel positions relative to the top left of the image at 0,0; *X* is the lateral direction, and *Y* is the longitudinal direction. To quantitatively evaluate tracking accuracy, we selected a random sample comprising 5% of the frames that were not used for AI training from the complete set of needle trail exercise video frames. We compared the positions of circles (AI detection results) and crosses (correct data) and found an average pixel discrepancy of 9.2.

## Qualitative evaluation

**Fig. 4:**
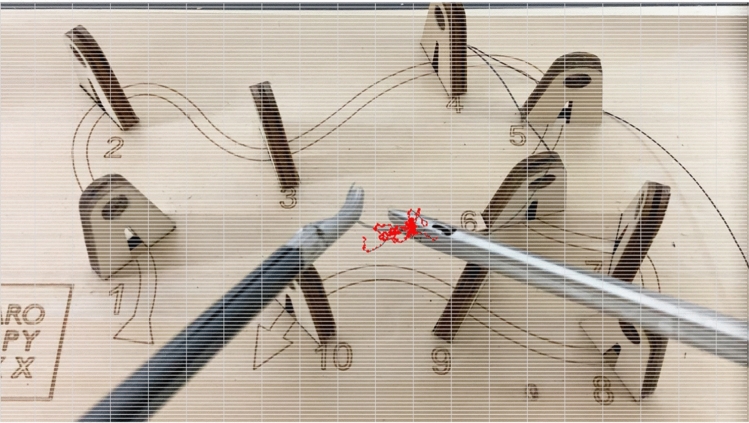
Results of tracking the movement of the right instrument, which was used to adjust the thread angle for 15 s

**Fig. 5:**
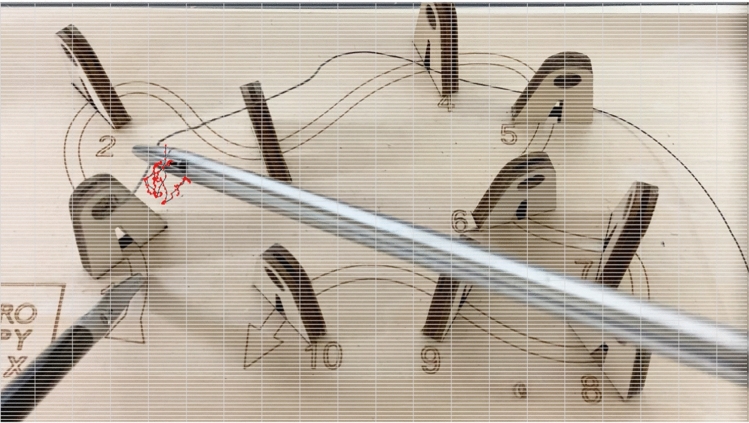
The results of tracking the movement of the right instrument, which was used to thread a needle through hole 1 on the training kit for 5 s

Figures 4 and 5 show the results of tracking the movement of tip1 of the right forceps. The input video has a resolution of 1980 pixels in height and 1080 pixels in width and a frame rate of 30/s. Figure 3 shows the movement of the right instrument, which was used to adjust the angle of a thread using both left and right instruments for 15 s. A red circle indicates the position of tip1 in each frame, and black lines connect these points. Figure 4 shows the results of tracking the movement of the right instrument, which was used to thread a needle through hole 1 on the training kit for 5 s. It is anticipated that there will be differences in stability (e.g., less shaking), and the time required to complete the task between skilled and unskilled practitioners when adjusting the thread angle. In addition, distinctive movements indicating hesitation in trajectory or retries can be observed when threading the needle.

### Evaluation limitations

Three primary factors were identified that contributed to instability in forceps detection:

1. Unobservability of the keypoints

We noted several circumstances that led to detection instability. These included instances where objects on the training kit obscured the keypoint or when the rotation of the forceps rendered the keypoint unobservable. Furthermore, we found that rapid movements, such as severe shaking, could cause the keypoint to become unobservable, which affects detection reliability.

2. Insufficient keypoint visual features

Our findings revealed that the keypoints, *tip1*, *tip2*, *tip3*, *stick1*, and *stick4* (Fig. 1), could be tracked with relative stability. However, instances of detection misalignment were frequently observed with *stick2* and *stick3*. We hypothesize that this is because *stick2* and *stick3* possess fewer visual features than the other keypoints and the proximity of their visual features and labeling positions. Detection instability was also observed when the visual features of the keypoints differed from the labeling because of reflective interference from the metal components.

3. Discrepancies between backgrounds during labeling and tracking

**Fig. 6:**
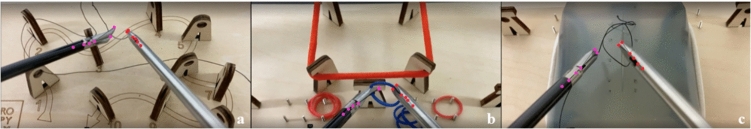
Three types of backgrounds used for evaluation. **a** Needle trail exercises. **b** Ring transfer exercises. **c** Suturing exercises

In this study, three types of backgrounds were incorporated into the evaluation process (Fig. 6). The training dataset was assembled from the video shown on the left in Fig. 6a. The accuracy of machine learning models is significantly enhanced when the training data closely resemble the actual inference data. Therefore, stable tracking was feasible when the video from Fig. 6a was used as input. However, the detection stability experienced a considerable decrease at the site of the orange and blue strings in Fig. 6b and in the vicinity of the pale blue object depicted in Fig. 6c.

## Discussion

Training for improving laparoscopic skills is essential for young pediatric surgeons. Simulator training is essential for young surgeons because it is difficult to master surgical techniques even with standard surgery [7, 8]. The most widely used laparoscopic skills training program is the Fundamentals of Laparoscopic Surgery (FLS), introduced in 2004 by the Society of American Gastrointestinal Endoscopic Surgeons as a laparoscopic surgical training program unique to laparoscopic surgery [13]. Since introducing the FLS program, surgical training using simulators has spread widely. However, the FLS program does not have a feedback system. Feedback on laparoscopic surgical skills is important to improve laparoscopic surgery [14]. Educational feedback is a crucial element of simulation-based surgical education, even in a controlled environment [15]. Therefore, we examined and reported the learning effects of our disease-specific laparoscopic surgical simulator on forceps operation techniques during continuous laparoscopic surgical skill training [11]. However, current methods for providing educational feedback tend to be subjective and not quantitative.

AI-based simulator training makes it possible to provide objective feedback and create an environment in which the same quality of feedback can be received anywhere [16]. Certification of basic laparoscopic skills can be provided anywhere in the world without the need for expert evaluators to perform the assessment [11]. It is expected that the simulator could delineate and quantitatively analyze the variations in forceps manipulation between novice learners and proficient users, assuming identifiable and measurable differences.

In this study, we used DLC to recognize and positionally measure the forceps in the operating field. DLC has been widely used to analyze biological behavior and tool movements, and its reliability is well established [12]. Alternative methods for positional measurements include attaching printed tags to the forceps or using generic models [17]. However, the method of attaching tags is not preferable because it alters the use of forceps in the surgical environment. Additionally, although large-scale multimodal models have been introduced, their reliability in positional measurement remains questionable. Therefore, in this study, task-specific AI models, such as DLC, that do not require physical tag attachment are considered more suitable.

We aim to continue improving the AI-based forceps manipulation evaluation system investigated in this study and bring new possibilities to surgical education using simulators. A plausible strategy for augmenting the stability of the tracking process entails the use of joint positional data of specific stable keypoints, such as *tip1–tip3*, and select keypoints on the stick. These keypoints demonstrated a consistent level of stability throughout the study. Considering the rigid structure of the forceps, substantial alterations in the interrelationships between the positions of these stable keypoints are improbable. Consequently, if a detected keypoint appears in an extensively distorted position, it could be categorized as a false detection. In addition, when a single keypoint becomes obscured by an object within the training kit, the position of the concealed keypoint can be inferred using the positional data of other visible keypoints. This strategy can maintain tracking stability even in the presence of transient obstructions. A comprehensive strategy incorporating individual backgrounds as the training dataset could be used to address the variability in detection accuracy across diverse backgrounds within the training kit. This approach is expected to mitigate the issues associated with detection accuracy that arise from variability in training kit backgrounds.

This study has several limitations. First, in quantitative evaluation, a pixel discrepancy of 9.2 is not a favorable result during a surgical procedure. Therefore, AI models can improve their accuracy via self-learning. It is necessary to self-learn in various situations for practical use. Second, rapid instrument movements reduce tracking accuracy. In addition, tracking accuracy was reduced due to rapid instrument movements, and image blurring was observed. Several techniques can be employed to address this issue. First, image sensors that capture images at higher frames per second can effectively reduce blurring in fast-moving scenes. Optimizing the lighting environment to ensure adequate illumination can help minimize blurring. Additionally, employing cameras with large image sensors that can capture clear images even under low light conditions is beneficial. Furthermore, instead of color image sensors, hardware, such as event cameras that specialize in visualizing differences and detecting fine movements, can be considered. Third, the accuracy of the measurements does not directly depend on the degree of experience of the participants but varies depending on how the forceps are manipulated. If the movements of the forceps by an expert are faster, the measurement accuracy may decrease due to image blurring. However, measurement accuracy can improve when the forceps are moved more carefully and efficiently. Therefore, the stability of the measurement accuracy is influenced by how the forceps are handled rather than the level of expertise.

Nevertheless, this research has potential. Tracking the movements of medical devices or instruments and linking the results to AI analysis are feasible. For instance, visualizing and accumulating the movements of devices used by many surgeons as graphs can allow the analysis of these data to quantitatively demonstrate the differences between the movements of experienced and inexperienced practitioners. Furthermore, these movements can be tracked, and the use of large-scale multimodal models (LMM) to verbalize the movements of surgical instruments (such as the opening and rotation of forceps) can enable inexperienced practitioners to qualitatively understand the differences between the processes performed by experts and novices.

A limitation of this research is that if each expert has a unique way of moving the instruments, there may be no statistical trends in the experts’ movements, potentially failing to generate useful data for novices. In addition, when using LMMs for analysis, there is a technical limitation in that the models lack specialized medical knowledge and, therefore, cannot provide expert explanations or advice. Addressing this requires methods such as transfer learning to impart AI with specialized knowledge.

Additionally, the AI-based posture estimation method in this study specializes in tracking the tips of forceps; however, using the same process as that used to train the features of other medical devices or instruments, it can also track these targets. In this study, training using images of forceps was conducted manually, but by employing similar techniques to track-specific points in the images continuously, it is possible to efficiently gather training images for other medical devices or instruments.

This study explored the feasibility of an AI model for quantifying forceps movement within a surgical training apparatus. In conclusion, improvements in the AI system and self-learning are expected to enable it to accurately distinguish between expert and novice surgeons. Our next goal is to further develop an AI-based skill assessment system for training, establish an efficient and reliable training environment for laparoscopic surgical techniques, and improve the safety of pediatric laparoscopic surgery.

## References

[1] Holcomb GW III, Olsen DO, Sharp KW (1991) Laparoscopic cholecystectomy in the pediatric patient. J Pediatr Surg 26:1186–1190. 10.1016/0022-3468(91)90330-v 1838118 10.1016/0022-3468(91)90330-v

[2] Rothenberg SS (2005) Thoracoscopic repair of esophageal atresia and tracheo-esophageal fistula. Semin Pediatr Surg 14:2–7. 10.1053/j.sempedsurg.2004.10.020 15770583 10.1053/j.sempedsurg.2004.10.020

[3] Velanovich V (2000) Laparoscopic vs open surgery: a preliminary comparison of quality-of-life outcomes. Surg Endosc 14:16–21. 10.1007/s004649900003 10653229 10.1007/s004649900003

[4] Varas J, Mejía R, Riquelme A, Maluenda F, Buckel E, Salinas J, Martínez J, Aggarwal R, Jarufe N, Boza C (2012) Significant transfer of surgical skills obtained with an advanced laparoscopic training program to a laparoscopic jejunojejunostomy in a live porcine model: feasibility of learning advanced laparoscopy in a general surgery residency. Surg Endosc 26:3486–3494. 10.1007/s00464-012-2391-4 22733192 10.1007/s00464-012-2391-4

[5] Boza C, León F, Buckel E, Riquelme A, Crovari F, Martínez J, Aggarwal R, Grantcharov T, Jarufe N, Varas J (2017) Simulation-trained junior residents perform better than general surgeons on advanced laparoscopic cases. Surg Endosc 31:135–141. 10.1007/s00464-016-4942-6 27139703 10.1007/s00464-016-4942-6

[6] Belmar F, Gaete MI, Escalona G, Carnier M, Durán V, Villagrán I, Asbun D, Cortés M, Neyem A, Crovari F, Alseidi A, Varas J (2023) Artificial intelligence in laparoscopic simulation: a promising future for large-scale automated evaluations. Surg Endosc 37:4942–4946. 10.1007/s00464-022-09576-1 36192656 10.1007/s00464-022-09576-1 PMC9529161

[7] Obata S, Ieiri S, Uemura M, Jimbo T, Souzaki R, Matsuoka N, Katayama T, Hashizume M, Taguchi T (2015) An endoscopic surgical skill validation system for pediatric surgeons using a model of congenital diaphragmatic hernia repair. J Laparoendosc Adv Surg Tech A 25:775–781. 10.1089/lap.2014.0259 26375773 10.1089/lap.2014.0259

[8] Jimbo T, Ieiri S, Obata S, Uemura M, Souzaki R, Matsuoka N, Katayama T, Masumoto K, Hashizume M, Taguchi T (2017) A new innovative laparoscopic fundoplication training simulator with a surgical skill validation system. Surg Endosc 31:1688–1696. 10.1007/s00464-016-5159-4 27519591 10.1007/s00464-016-5159-4

[9] Fukuta A, Obata S, Jimbo T, Kono J, Souzaki R, Matsuoka N, Katayama T, Taguchi T (2019) Continuous skill training using the disease-specific endoscopic surgical simulator to promote young pediatric surgeons: learning curve for trainees. J Laparoendosc Adv Surg Tech 29:1334–1341. 10.1089/lap.2019.0111 10.1089/lap.2019.0111 31313947

[10] Munro C, Burke J, Allum W, Mortensen N (2021) COVID-19 leaves surgical training in crisis. BMJ 372:n659. 10.1136/bmj.n659 33712499 10.1136/bmj.n659

[11] Fazlollahi AM, Bakhaidar M, Alsayegh A, Yilmaz R, Winkler-Schwartz A, Mirchi N, Langleben I, Ledwos N, Sabbagh AJ, Bajunaid K, Harley JM, Del Maestro RF (2022) Effect of artificial intelligence tutoring vs expert instruction on learning simulated surgical skills among medical students: a randomized clinical trial. JAMA Netw Open 5:e2149008. 10.1001/jamanetworkopen.2021.49008 35191972 10.1001/jamanetworkopen.2021.49008 PMC8864513

[12] Mathis A, Mamidanna P, Cury KM, Abe T, Murthy VN, Mathis MW, Bethge M (2018) DeepLabCut: markerless pose estimation of user-defined body parts with deep learning. Nat Neurosci 21:1281–1289. 10.1038/s41593-018-0209-y 30127430 10.1038/s41593-018-0209-y

[13] Peters JH, Fried GM, Swanstrom LL, Soper NJ, Sillin LF, Schirmer B, Hoffman K, Committee SAGESFLS (2004) Development and validation of a comprehensive program of education and assessment of the basic fundamentals of laparoscopic surgery. Surgery 135:21–27. 10.1016/s0039-6060(03)00156-9 14694297 10.1016/s0039-6060(03)00156-9

[14] Singh P, Aggarwal R, Tahir M, Pucher PH, Darzi A (2015) A randomized controlled study to evaluate the role of video-based coaching in training laparoscopic skills. Ann Surg 261:862–869. 10.1097/SLA.0000000000000857 25185469 10.1097/SLA.0000000000000857

[15] Issenberg SB, McGaghie WC, Petrusa ER, Lee Gordon D, Scalese RJ (2005) Features and uses of high-fidelity medical simulations that lead to effective learning: a BEME systematic review. Med Teach 27:10–28. 10.1080/01421590500046924 16147767 10.1080/01421590500046924

[16] Uemura M, Tomikawa M, Miao T, Souzaki R, Ieiri S, Akahoshi T, Lefor AK, Hashizume M (2018) Feasibility of an AI-based measure of the hand motions of expert and novice surgeons. Comput Math Methods Med 2018:9873273. 10.1155/2018/9873273 29686724 10.1155/2018/9873273 PMC5857335

[17] Tsui D, Ramos K, Melentyev C, Rajan A, Tam M, Jo M, Ahadian F, Talke FE (2024) A low-cost, open-source-based optical surgical navigation system using stereoscopic vision. Microsyst Technol. 10.1007/s00542-024-05668-1

