# Correction: Artificial intelligence facilitates the potential of simulator training: An innovative laparoscopic surgical skill validation system using artificial intelligence technology

**DOI:** 10.1007/s11548-024-03300-1

**Published:** 2024-12-12

**Authors:** Atsuhisa Fukuta, Shogo Yamashita, Junnosuke Maniwa, Akihiko Tamaki, Takuya Kondo, Naonori Kawakubo, Kouji Nagata, Toshiharu Matsuura, Tatsuro Tajiri

**Affiliations:** 1 Department of Pediatric Surgery, Reproductive and Developmental Medicine, Faculty of Medical Sciences, Kyushu University, 3-1-1 Maidashi, Higashi-ku, Fukuoka, 812-8582 Japan; 2 Exawizards Inc., Tokyo, Japan

**Correction to: International Journal of Computer Assisted Radiology and Surgery** 10.1007/s11548-024-03253-5

The article Artificial intelligence facilitates the potential of simulator training: An innovative laparoscopic surgical skill validation system using artificial intelligence technology, written by Atsuhisa Fukuta, Shogo Yamashita, Junnosuke Maniwa, Akihiko Tamaki, Takuya Kondo, Naonori Kawakubo, Kouji Nagata,Toshiharu Matsuura and Tatsuro Tajiri, was originally published electronically on the publisher’s internet portal on 19 August 2024 without open access. With the author(s)’ decision to opt for Open Choice the copyright of the article changed on 19 November 2024 to © The Author(s) 2024 and this article is licensed under a Creative Commons Attribution 4.0 International License, which permits use, sharing, adaptation, distribution and reproduction in any medium or format, as long as you give appropriate credit to the original author(s) and the source, provide a link to the Creative Commons licence, and indicate if changes were made. The images or other third party material in this article are included in the article’s Creative Commons licence, unless indicated otherwise in a credit line to the material. If material is not included in the article’s Creative Commons licence and your intended use is not permitted by statutory regulation or exceeds the permitted use, you will need to obtain permission directly from the copyright holder. To view a copy of this licence, visit http://creativecommons.org/licenses/by/4.0/.

The original article has been corrected.

